# Indigenous oyster fisheries persisted for millennia and should inform future management

**DOI:** 10.1038/s41467-022-29818-z

**Published:** 2022-05-03

**Authors:** Leslie Reeder-Myers, Todd J. Braje, Courtney A. Hofman, Emma A. Elliott Smith, Carey J. Garland, Michael Grone, Carla S. Hadden, Marco Hatch, Turner Hunt, Alice Kelley, Michelle J. LeFebvre, Michael Lockman, Iain McKechnie, Ian J. McNiven, Bonnie Newsom, Thomas Pluckhahn, Gabriel Sanchez, Margo Schwadron, Karen Y. Smith, Tam Smith, Arthur Spiess, Gabrielle Tayac, Victor D. Thompson, Taylor Vollman, Elic M. Weitzel, Torben C. Rick

**Affiliations:** 1 Temple University, Department of Anthropology, Philadelphia, PA USA; 2 San Diego State University, Department of Anthropology, San Diego, CA USA; 3 University of Oklahoma, Department of Anthropology, Norman, OK USA; 4 University of Oklahoma, Laboratories of Molecular Anthropology and Microbiome Research, Norman, OK USA; 5 National Museum of Natural History, Smithsonian Institution, Department of Anthropology, Washington, DC USA; 6 University of Georgia, Department of Anthropology, Athens, GA USA; 7 California Department of Parks and Recreation, Santa Cruz District, Felton, CA USA; 8 University of Georgia, Center for Applied Isotope Studies, Athens, GA USA; 9 Western Washington University, Environmental Science, Bellingham, WA USA; 10 Muscogee Nation, Department of Historical and Cultural Preservation, Okmulgee, OK USA; 11 University of Maine, School of Earth and Climate Sciences, Orono, ME USA; 12 University of Maine, Climate Change Institute, Orono, Maine, USA; 13 University of Florida, Florida Museum of Natural History, Gainesville, FL USA; 14 National Park Service, Southeast Archeological Center, Tallahassee, FL USA; 15 Department of Anthropology, University of Victoria, Victoria, BC Canada; 16 Monash University, Monash Indigenous Studies Centre, ARC Centre of Excellence for Australian Biodiversity & Heritage, Melbourne, VIC Australia; 17 University of Maine, Department of Anthropology, Orono, ME USA; 18 University of South Florida, Department of Anthropology, Tampa, FL USA; 19 Michigan State University, Department of Anthropology, East Lansing, MI USA; 20 South Carolina Department of Natural Resources, Heritage Trust Program, Columbia, SC USA; 21 University of Queensland, School of Social Science, Brisbane, QLD Australia; 22 Maine Historic Preservation Commission, Augusta, ME USA; 23 George Mason University, Department of History and Art History, Fairfax, VA USA; 24 University of Connecticut, Department of Anthropology, Storrs, CT USA; 25 Max Planck Institute for the Science of Human History, Department of Archaeology, Jena, Germany

**Keywords:** Sustainability, Archaeology, Conservation biology

## Abstract

Historical ecology has revolutionized our understanding of fisheries and cultural landscapes, demonstrating the value of historical data for evaluating the past, present, and future of Earth’s ecosystems. Despite several important studies, Indigenous fisheries generally receive less attention from scholars and managers than the 17th–20th century capitalist commercial fisheries that decimated many keystone species, including oysters. We investigate Indigenous oyster harvest through time in North America and Australia, placing these data in the context of sea level histories and historical catch records. Indigenous oyster fisheries were pervasive across space and through time, persisting for 5000–10,000 years or more. Oysters were likely managed and sometimes “farmed,” and are woven into broader cultural, ritual, and social traditions. Effective stewardship of oyster reefs and other marine fisheries around the world must center Indigenous histories and include Indigenous community members to co-develop more inclusive, just, and successful strategies for restoration, harvest, and management.

## Abstract (web summary)

‘Commercial fisheries have decimated keystone species, including oysters in the past 200 years. Here, the authors examine how Indigenous oyster harvest in North America and Australia was managed across 10,000 years, advocating for effective future stewardship of oyster reefs by centering Indigenous peoples.’

## Introduction

Conservation biology increasingly recognizes the interconnections between colonial atrocities against Indigenous peoples and contemporary environmental disasters, with several studies emphasizing the importance of the co-production of knowledge in setting conservation priorities and goals^1–5^. Despite significant advances, Indigenous knowledge and archaeology are often neglected in conservation and ecology, including in studies of shellfish like oysters (Ostreidae). Oysters are important components and indicators of resilient coastal ecosystems^6^, but they also carry cultural and economic significance for people worldwide. Their ecological and cultural roles became well-established when post-glacial sea level rise created and stabilized estuaries around the world^7^. Despite contributions from conservation paleobiology and archaeology^8–12^, oyster management strategies often rely primarily on knowledge and data gathered during the past 200 years or less, a period during which many oyster fisheries collapsed under the weight of over-harvest, pollution, competition with non-native species, and habitat loss^13–17^. Today, the decline of oyster fisheries is a global phenomenon^13,14,17,18^, with as much as 85% of 19^th^ century oyster reef area lost by the early 21^st^ century^18^.

In contrast to capitalist commercial fisheries, intensive Indigenous fisheries thrived for millennia. Although the term “commercial” is widely used in the ecological and fisheries literature, we add the term “capitalist” to recognize that some Indigenous fisheries shared aspects of commercial fisheries, such as surplus production, exchange with other groups, and accumulation of wealth. We build on historical ecological research by Kirby^15^, who focused on the 17th–20th century capitalist commercial oyster fisheries of North America (the Atlantic *Crassostrea virginica* [Gmelin] and the Pacific *Ostrea lurida* [Carpenter]) and eastern Australia (*Saccostrea glomerata* [Gould]). These regions have well-known ecological histories and high restoration potential. Moreover, they are located within settler colonial nations where European and Indigenous histories are closely intertwined. While research tends to focus on colonial and capitalist commercial oyster harvest, in many cases the displacement and exclusion of Indigenous peoples was a key precipitating factor that allowed the rise of capitalist commercial fisheries, watershed disturbances, and the disruption of local ecosystems. Today, oyster fisheries cannot be effectively restored and managed without considering the past, present, and future roles of Indigenous people. Ecosystem health, however, is only one consideration—the inclusion of Indigenous people’s voices in their ancestral ecosystems also restores some of the rights that they are deprived of under colonial governments.

Here we establish the cultural significance, intensity, duration, and extent of “forgotten” oyster fisheries of Indigenous communities, in the same regions considered by Kirby^15^, for more than 6000 years before the arrival of European colonists. Our analysis integrates regional sea level histories, quantitative archaeological oyster abundance data, descriptions of the size, function, and distribution of archaeological sites containing oysters, and ethnohistoric accounts of oyster harvest, management, and farming. Much of the data generated in this study come from oyster-bearing shell middens, archaeological sites with accumulations of shell, animal bone, plant remains, and other artifacts. Shell middens have often been described by archaeologists as domestic refuse deposits, but shell middens are complex, engineered spaces that range from small, sometimes seasonal sites to massive, intricately designed mounds and rings, often with deeply symbolic and ritual meaning for Indigenous peoples in the past and present^19–21^. The millennial-scale records we present here document resilient ecosystem dynamics between people and oysters, even under conditions of intensive harvest. While focused on oysters and estuarine ecosystems, our research provides a model for the investigation of human interactions with other organisms and ecosystems globally and on long time scales.

## Results

**Fig. 1:**
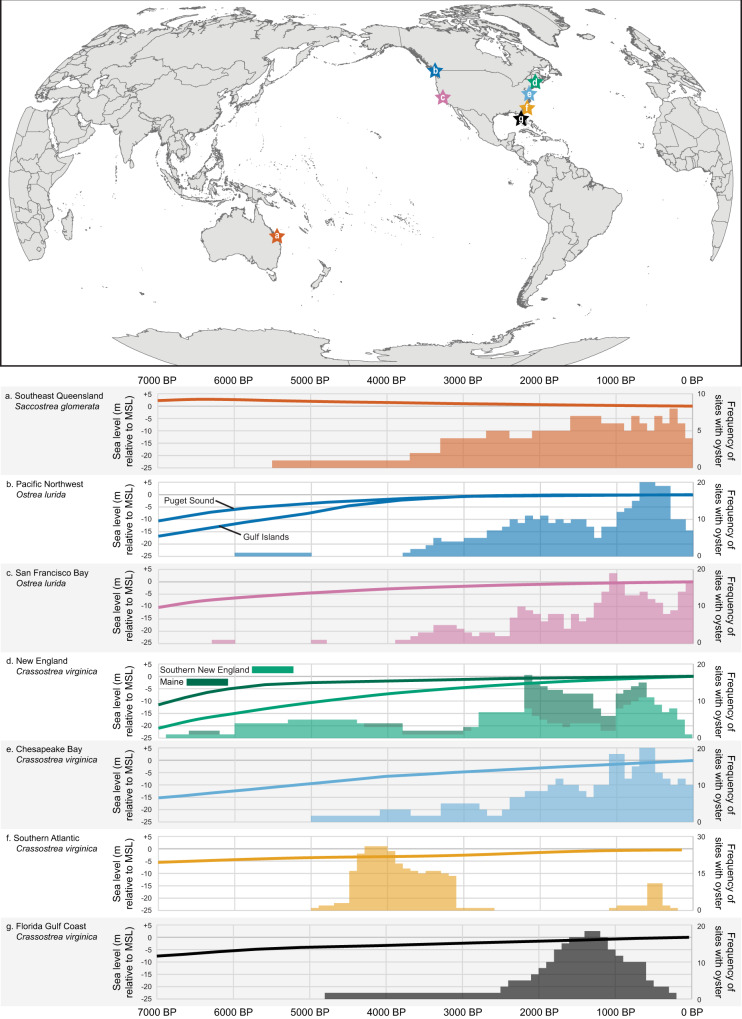
Oyster sites and sea level through time. Top map shows the location of each study region, while panels **a**–**f** show relative frequency of oyster bearing archaeological sites used for this study with sea level curves specific to each region. **a** Southeast Queensland (dark orange). **b** Pacific Northwest of North America (dark blue). **c** San Francisco Bay (pink). **d** New England (green). **e** Chesapeake Bay (light blue). **f** Southern Atlantic (light orange). **g** Peninsular Florida Gulf Coast (gray). Each site that contains oyster was counted once for each century during which it was likely occupied, based on radiometric dating or reliable artifact associations. Sea level curves were reproduced using data from Shugar and colleagues^26^ for the Pacific Northwest, Kelley and colleagues^23^ for Maine, Love and colleagues^25^ for Chesapeake Bay, Hawkes and colleagues^22^ for the southern Atlantic, and Khan and colleagues^24^ for the Gulf of Mexico in Florida. Sea level curves for southeast Queensland, San Francisco Bay, and southern New England were modeled for each region using the ICE_6G_C model^95^. MSL = modern mean sea level. Source data for oyster site frequency are provided as a Source Data file.

We focused on three regions: eastern Australia, the Pacific Coast of North America, and the Atlantic and Gulf of Mexico coast of North America to expand on previous historical studies^15^ (Fig. 1). These regions experienced thousands of years of oyster harvest by Indigenous peoples, followed by successive waves of dispossession of Indigenous peoples by colonial governments, widespread ecological change and overfishing, and, ultimately, the collapse of local oyster fisheries.

### Indigenous fisheries of abundance

The abundance of oyster-bearing archaeological sites speaks to the scale of Indigenous oyster fisheries, the long-term entanglement between fisheries and ecosystems, and the importance of oysters in Indigenous diet and culture. Initially, Indigenous oyster fisheries in these regions were constrained by sea level change and the establishment of estuaries primarily during the Early (11,700–8235 b2k, or before 2000 CE) and Middle (8235–4250 b2k) Holocene (Fig. 1). Sea level in northern North America is particularly variable because of the proximity to continental ice sheets, which created isostatic effects that combined with global sea level rise in complex ways. Despite this local variability, relative sea level in all of our North American study areas was rising by the Middle Holocene and was near modern by the Late Holocene (4250 BP to present)^22–26^. In Australia, on the other hand, a sea level high stand ~1.19 m above modern mean sea level (MSL) around 6900 BP was followed by a gradual decline to modern sea level over the past 2000–3000 years^27^. Sea level stabilization allowed estuaries, which are necessary habitat for many oyster species, to expand and set the stage for intensive oyster exploitation. However, site visibility, preservation, and discovery, and histories of human population growth, settlement patterns, and resource use were also important in structuring the archaeological record of oyster-bearing sites^28–30^. Although there is evidence of oyster and other estuarine shellfish harvest on California’s Northern Channel Islands ~11,500 years ago^31,32^, in our study regions the earliest known intensive oyster harvest occurs after sea level rise slowed during the Middle Holocene, with increasing numbers of oyster-bearing sites throughout the Late Holocene until the onset of colonialism or the displacement of Indigenous people (Fig. 1).

**Fig. 2:**
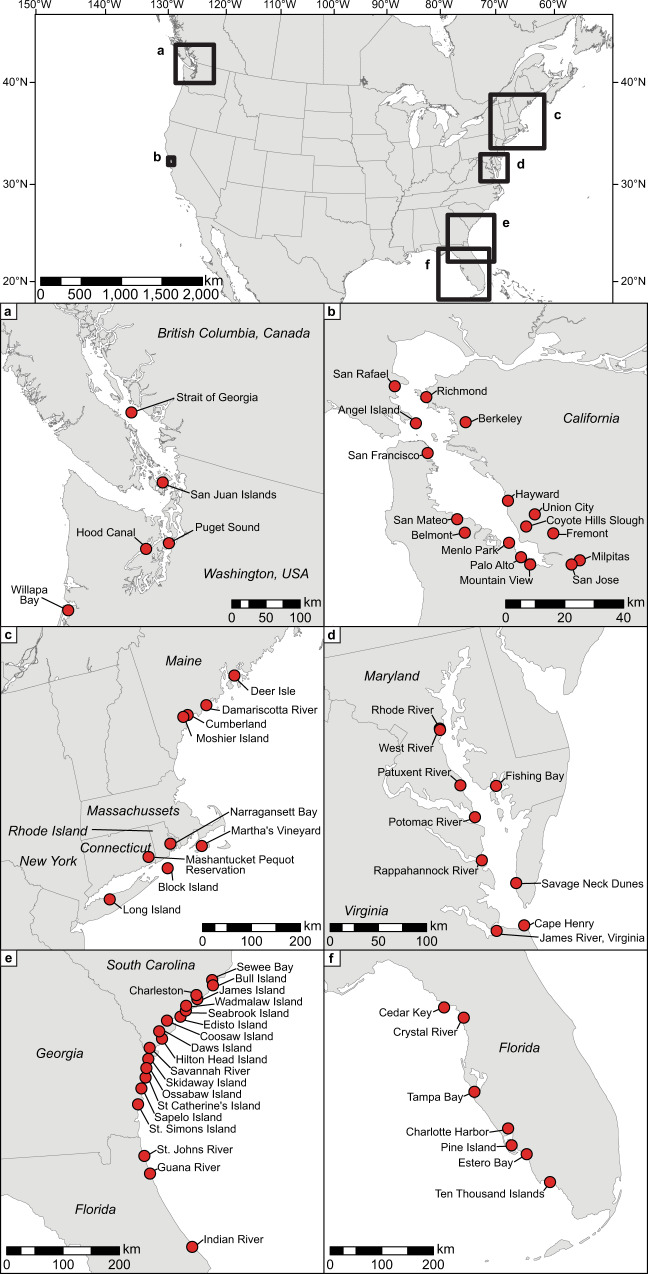
Maps of North American study areas. Locations of oyster-bearing sites used in this study, including **a** The Pacific Northwest of North America, **b** San Francisco Bay, **c** New England, **d** Chesapeake Bay, **e** The Atlantic coast of the southeastern US, and **f** The Peninsular Gulf Coast of Florida. Red circles represent more specific locations and correspond to site locations in Supplementary Data 1.

The exceptions to this pattern occur in the eastern U.S. The intensive oyster fishery of the southeastern U.S. occurred relatively early, during the end of the Middle Holocene and early part of the Late Holocene, followed by a diminished use of oysters for over a millennium in some areas. Once oyster harvest resumed in force in this region, there were considerably more oyster-bearing sites, reflecting an overall increase in all kinds of archaeological sites. However, few of these individual sites reached the size, extent, and density of oyster relative to the earlier shell mounds in the area, perhaps because of a focus on maize agriculture that increased in some areas just before European arrival. Along Peninsular Florida’s Gulf Coast, oyster exploitation peaked between 2000–1000 BP. Although, our data indicate a decline in harvesting of oysters before the arrival of Europeans, this likely occurred after their arrival. The data presented here reflect, in part, the difficulty of sampling and dating deposits just prior to European arrival. On the other hand, the intensive use of oysters in parts of Maine, especially along the Damariscotta River (Fig. 2), does appear to have ended several centuries before European arrival, perhaps because of shifting environmental conditions^33^.

**Fig. 3:**
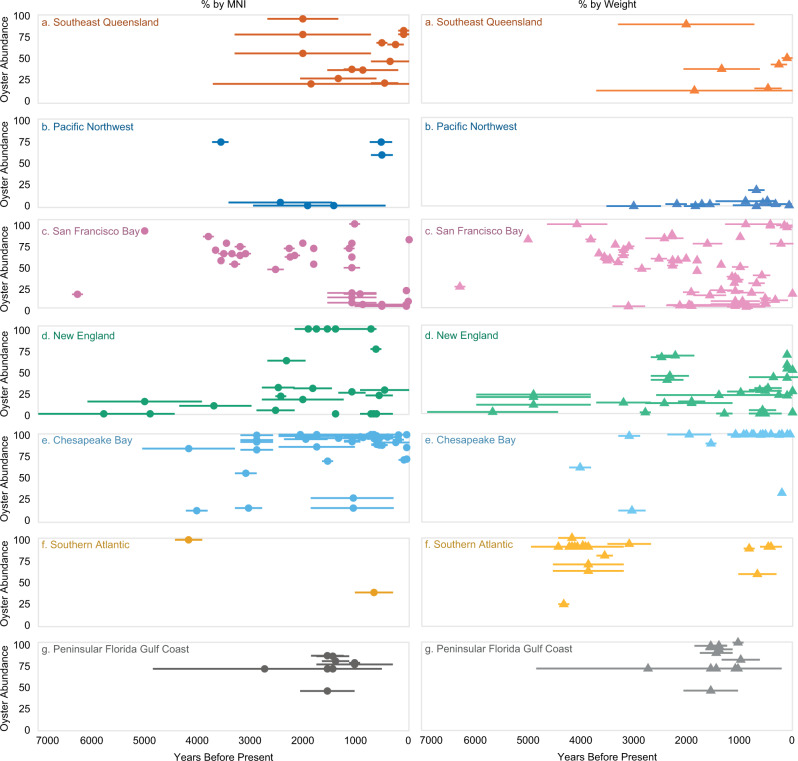
Relative abundance of Oyster within sites. Some studies report minimum number of individuals (MNI, left panel) and others report weight (right panel). Although these are not directly comparable, both measurements provide valuable information about relative abundance. Each line represents one site. Its position on the Y-axis represents the relative proportion of oyster in the site and its length on the X-axis represents the estimated period of time that a site was occupied (X-axis). The midpoint symbol helps differentiate overlapping sites. Panel letter and color designations correspond with the map in Fig. 1. **a** Southeast Queensland (dark orange). **b** Pacific Northwest of North America (dark blue). **c** San Francisco Bay (pink). **d** New England (green). **e** Chesapeake Bay (light blue). **f** Southern Atlantic (light orange). **g** Peninsular Florida Gulf Coast (gray). See Table S1 for details and references. Source data are provided as a Source Data file.

Oysters are most abundant in sites on the east coast of North America, where estuaries today are more extensive than along the Pacific Coast (Fig. 3). Along the coast of the southeastern United States, oysters make up over half of the shellfish in all but a few archaeological sites, and oysters account for at least 90% of total shellfish weight at 60% of our study sites (Fig. 3). Along Chesapeake Bay, oysters typically dominate shell middens to the near exclusion of other shellfish, with 26 of 30 sites containing more than 90% oyster by weight. In New England, people relied less on oysters in favor of hard shell clam (*Mercenaria mercenaria* Linnaeus) and soft shell clam (*Mya arenaria*) (Fig. 3). The only New England sites in our analysis where oysters account for more than 90% of shellfish by weight are the large Late Holocene shell middens along Maine’s Damariscotta River (Fig. 2).

On the Pacific Coast of North America, people used oysters very differently. This is partly the result of coastal geomorphology, with fewer large estuaries along the Pacific Coast than the Atlantic Coast of North America. Along the Northwest Coast, oysters provided a relatively small supplement to diverse diets of fish and shellfish, including species like butter and littleneck clams that were abundant and dried and stored^34,35^. The distribution of Northwest Coast sites with high oyster abundance is patchy and spatially associated with deltaic microenvironments. In San Francisco Bay, the emphasis on oyster use changed through time, possibly as oyster populations fluctuated in response to climate change. The general long-term trend in oyster use in the San Francisco Bay area is one of increases and decreases in oyster remains, with oyster representing as much as 94% to as low as 2% of shellfish weight (Fig. 3 and Table S1). In general, Pacific Coast sites show high levels of resource diversity. People harvested a wide variety of aquatic and terrestrial foods, incorporating oysters periodically as a dense, predictable resource.

Estuarine shell middens in southeastern Queensland, Australia, also have a high degree of variability in oyster abundance, although lower overall resource diversity reflects targeted harvesting of oysters at some sites. These sites are composed primarily of shellfish, with few vertebrate remains. Oysters were always an important component of First Nations people’s shellfish consumption in this part of Australia, contributing at least 20% and as much as 95% of the total estuarine shellfish by weight (Fig. 3).

### Oysters in monuments and ritual landscapes

**Fig. 4:**
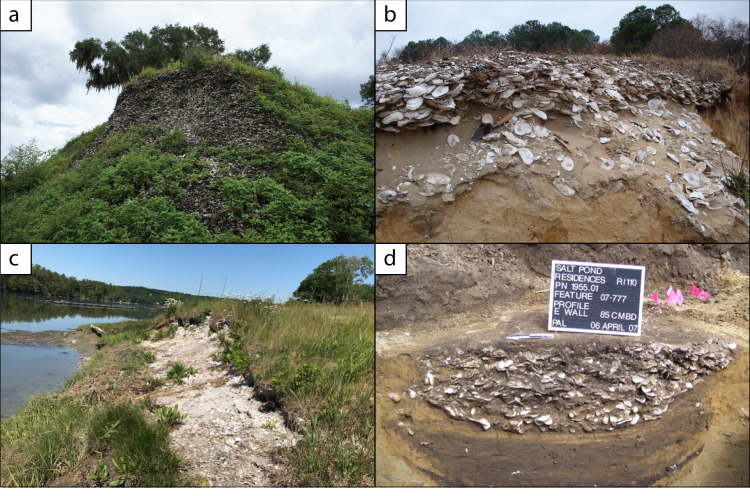
Examples of archaeological sites, North American Atlantic and gulf coasts. **a** Mound A made almost entirely of oyster at the Crystal River site, Florida Gulf Coast, which is estimated to contain 20–30 million oysters which accumulated between 1330-800 years ago (Photo credit: Victor Thompson). **b** A small Late Holocene site at Fishing Bay, Maryland, perhaps a camp or processing site, with some 2 million oysters which accumulated from 1250–950 years ago (Photo credit: Torben Rick). **c** Oyster shell midden dated to 2200–1300 years ago at Site 26.15 on the Damariscotta Estuary, Maine (Photo credit: Bonnie Newsom). **d** Small pit feature with oysters and other food remains on the coast of Rhode Island dated to 1000–500 years ago (Late Woodland) (Photo credit: Kevin McBride).

**Fig. 5:**
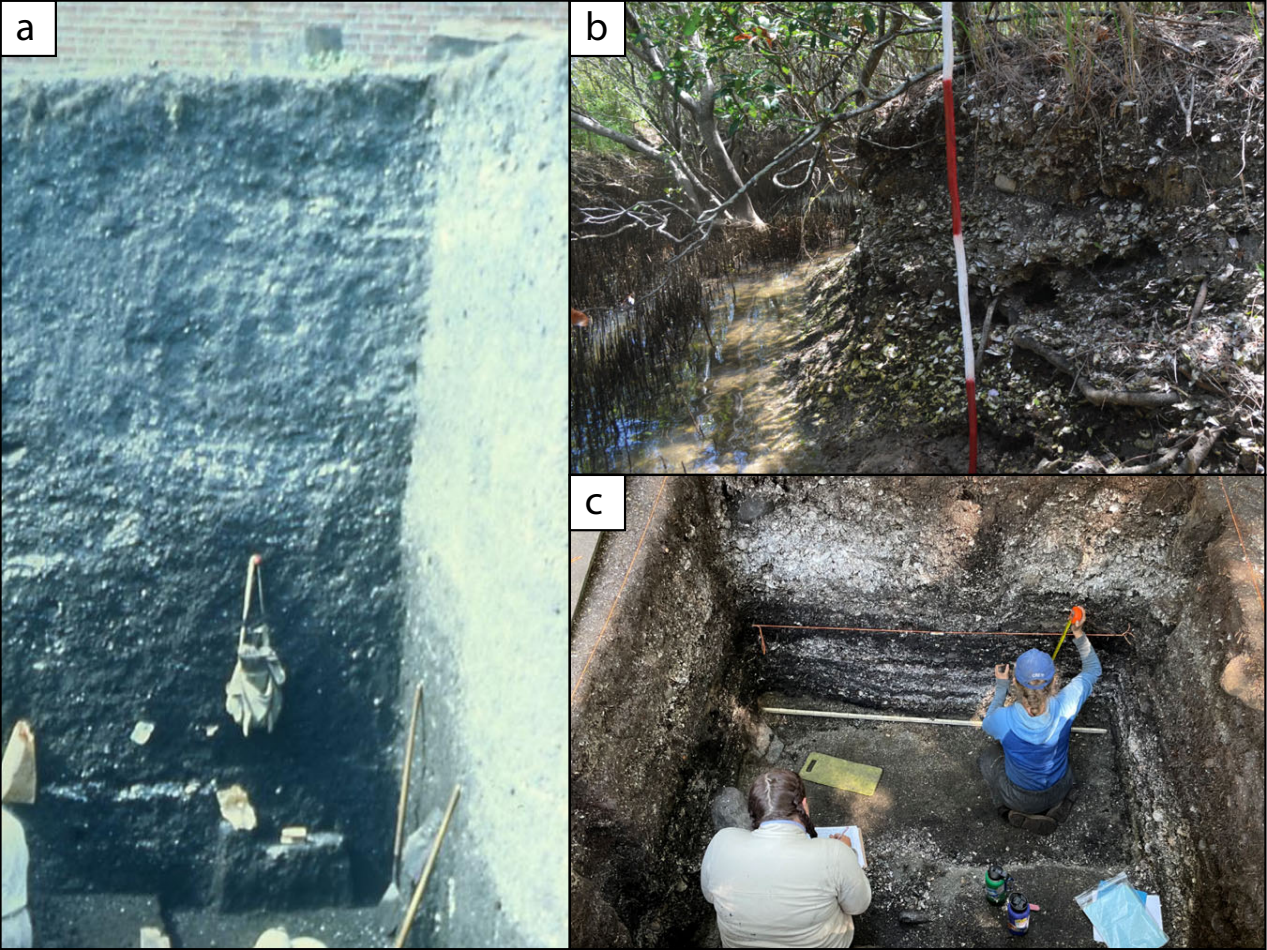
Examples of archaeological sites, Pacific coast of North America and Australia. **a** Profile of the West Berkeley Shell Mound, California exposed during excavation. This massive site dates from 6000 years ago through about 1100 years ago and is composed of oysters and other materials and also contains evidence for ceremonialism and ritual (Photo credit: Phoebe Hearst Museum of Anthropology, University of California Berkeley). **b** Bli Bli Midden, Southeast Queensland, showing dense shellfish concentrations, including oysters and dating to before 1850 (Photo credit: Kate Greenwood. Courtesy of the Kabi Kabi Peoples Aboriginal Corporation). **c** Dense shell midden deposit spanning the past 1000 years as exposed during excavation at a Tseshaht First Nation village in the Pacific Northwest (Photo credit: Iain McKechnie).

While environmental conditions and food choices are important factors in understanding ancient Indigenous oyster harvest, they tell only part of the story. Oyster data in this study come from a wide range of archaeological sites representing diverse human activities from food consumption to rituals to the construction of mounded and monumental architecture. These sites also contain information about how the intensity of past oyster harvest figured into larger cultural, social, and political dynamics. For example, some archaeological sites in this study (Table S1) were small temporary camps perhaps used only for a season or two. At other sites, oysters were part of ritualized monumental constructions that served as important political or social symbols^21,33,36,37^ or used in the construction of anthropogenic islands and elaborate water control features for fish storage^38^ (Figs. 4 and 5).

Large structural mounds and other sites made primarily of shells indicate that oysters and other shellfish were closely connected to Indigenous peoples’ social, political, and ritual lives. They also demonstrate the scale and intensity of oyster harvest in these regions. In the southeastern U.S., for example, Native Americans have a history of constructing massive mounds of earth and shell in coastal and riverine areas dating back over 5000 years^39^ (Fig. 4). At the Crystal River site on Peninsular Florida’s Gulf Coast (Fig. 2), Mound A is just one of six mounds built over the course of about 600 years. It covered at least 9000 m^2^, even after approximately 1/3 of the mound was destroyed during the historical period. At its highest point, the mound stood ~9 m above the surrounding landscape, flattened at the top to create a platform for ritual activities^37^. The extant part of this mound may have contained nearly 20 million oysters, and it could have contained some 30 million oysters before it was partially destroyed (Table 1). Similarly, Maine’s Whaleback Shell Midden was at least 6 m deep, covered an area at least 105 m long and 37 m wide, and was made almost entirely of oyster shell^33^. In San Francisco Bay, shell mounds like the West Berkeley mound were massive sites composed almost entirely of shell including oyster. They also contained Indigenous ancestors’ burials, which indicate a combination of ritual, ceremonial, and feasting activities, and household subsistence^40,41^(Fig. 5).

**Table 1:** Estimated absolute abundance of oysters.

Region	Site	Estimated Site Volume (m^3^)	Site Mapping Method	Excavated Volume (m^3^)	Oyster MNI	Estimated total Oysters in Site
Southeast Queensland	Booral Shell Mound Square A & B	216	Surface observations & excavation	0.5	14639	5,955,035
Southeast Queensland	Tin Can Inlet Midden Site 62, Square B & C	34	Surface observations & excavation	0.1	415	166,000
Southeast Queensland	White Patch Site 3	9	Surface observations & excavation	0.1	169	11,830
Southeast Queensland	St Helena Island Site	7500	Surface observations & excavation	0.1	949	50,123,239
New England	Whaleback	4360	Excavation	4200.0	–	–
New England	Hornblower II	173	Surface observations & excavation	0.3	196	130,642
New England	Pratt	156	Surface observations & excavation	0.2	1268	1,039,093
New England	Cunningham	963	Surface observations & excavation	0.1	8	96,280
Chesapeake Bay	18AN226	1324	Surface observations & excavation	0.1	840	22,238,160
Chesapeake Bay	18AN285	10080	Surface observations & excavation	1.1	286	2,620,800
Chesapeake Bay	18AN286	86	Surface observations & excavation	0.6	568	84,612
Chesapeake Bay	18AN287	176	Surface observations & excavation	0.5	334	114,935
Chesapeake Bay	18AN308	614	Surface observations & excavation	0.7	157	130,321
Chesapeake Bay	18AN1323	19	Surface observations & excavation	0.1	339	106,785
Chesapeake Bay	18DO35	1050	Surface observations & excavation	0.0	77	4,755,882
Chesapeake Bay	18DO127	4781	Surface observations & excavation	0.0	313	46,766,602
Chesapeake Bay	18DO130	1	Surface observations & excavation	0.1	104	1,560
Chesapeake Bay	18DO130	100	Surface observations & excavation	0.1	200	400,000
Chesapeake Bay	18DO429	1250	Surface observations & excavation	0.1	94	2,350,000
Chesapeake Bay	18DO436	750	Surface observations & excavation	0.1	202	2,020,000
Chesapeake Bay	18DO439	420	Surface observations & excavation	0.0	186	2,367,273
Chesapeake Bay	44NH478	150	Surface observations & excavation	0.1	85	127,500
Southeastern US–Atlantic Coast	Sea Pines	833	LiDAR & total station mapping	2.3	436	161,475
Southeastern US–Atlantic Coast	Pockoy Island 1	2046	LiDAR & total station mapping	0.7	480	1,327,070
Southeastern US–Atlantic Coast	Fig Island 2	11643	LiDAR & total station mapping	0.7	4548	75,645,585
Peninsular Florida Gulf Coast	Roberts Island	6971	LiDAR & total station mapping	1.0	2821	19,665,191
Peninsular Florida Gulf Coast	Cockroach Key	28618	LiDAR & total station mapping	0.3	8085	925,515,822
Peninsular Florida Gulf Coast	Safety Harbor	23371	LiDAR & total station mapping	0.3	753	70,393,452
Peninsular Florida Gulf Coast	Mound Key (8LL2)	603390	LiDAR & total station mapping	0.3	7714	18,618,192,583
Peninsular Florida Gulf Coast	Shell Mound	29310	LiDAR & total station mapping	0.0	1018	2,131,241,171

This subsample of sites reported sufficient data, including estimated site volume and minimum number of individual (MNI) oysters within a known excavated volume.

In other areas, oysters were deposited primarily in horizontal layers of shell, animal bone, plant remains, artifacts, and other materials. Although these lacked the physical scale of monumental shell mounds, they formed durable and symbolically infused features that persisted on the landscape for centuries or more. In the Pacific Northwest, large sites that contained oyster among other food remains were often associated with villages and settlements occupied for millennia (Fig. 5). These comprised the remains of daily and ceremonial food in large-scale terraformed site deposits^36^. In Australia, southeastern Queensland sites range from large, dense deposits and low mounds to smaller sites with evidence for a wide range of subsistence foods and some sites with very intensive oyster harvest, many of which were part of complex patterns of land use, ritual, and ceremony. The Booral Shell Mound on the mainland coast opposite K’gari (Fraser Island) stood ~1.4 m high and had a footprint of about 154 m^2^,^42^. Even at this relatively small mound where oyster was one of several important shellfish, people harvested approximately 5.9 million oysters (Table 1).

Each of these areas also contains smaller camps or sites with shorter-term occupations, a pattern typified by site 18DO439 in Chesapeake Bay. This Late Holocene site was likely used for a few centuries or less by small groups of people who came primarily to harvest oysters^43^. Covering just 1400 m^2^, we estimate that over 2.3 million oysters were deposited (Table 1 and Fig. 4). Even smaller still are archaeological pit features, which were small pits for storage or refuse often covering an area only 1–2 m in diameter (Fig. 4). Although they are small, these features are ubiquitous along the east coast of North America and many other parts of the world, the result of a sustained intensive harvest that persisted for millennia^10,11^. These archaeological sites illustrate the connections between the oyster harvest, traditional knowledge, and the ritual, ceremonial, and cultural patterns of Indigenous peoples.

### Not all forgotten: Indigenous use of oysters

Indigenous oral histories and ethnohistoric or ethnographic accounts provide records of people’s relationships with oysters worldwide, such as the Seri in Sonora, Mexico who collected oysters from offshore reefs^20^. Available accounts were often recorded at a time when many Indigenous peoples were being displaced from their traditional homelands and devastated by introduced disease, massacres, and sustained violence, but still illustrate broad connections between people and oysters.

**Fig. 6:**
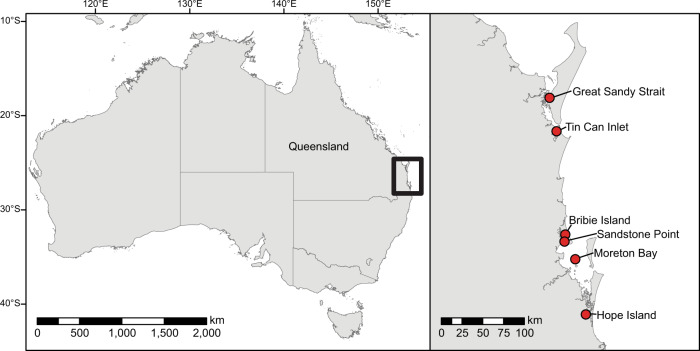
Map of Australian study area, Southeast Queensland. Locations of oyster-bearing sites correspond to site locations in the Supplementary Data 1.

In southeastern Queensland, Australia, accounts from the 1840s and 1860s indicate that oysters were eaten raw and roasted^44^, canoes were used to travel to islands to harvest oysters that were placed in a fire to cleanse and stew them^45^, and oysters were harvested at low tides with people carrying spears and using dilly bags^46^. Today, the Quandamooka Aboriginal people of Moreton Bay (Fig. 6) extend the seasonal availability of oysters ‘using techniques handed down from their ancestors’^47^. On natural high points on the shallow seabed, they construct artificial reefs using old oyster shells to catch oyster spat. Depleted reefs are restocked with young ‘farmed’ oysters to ensure sustainable harvests throughout the year. Oyster farming and cultivation likely happened, but has been difficult to identify in the archaeological record^48^. The challenge of recognizing specific archaeological correlates for oyster management also persists in other regions, although the sustainable nature of harvest over thousands of years demonstrates that people had powerful traditional ecological knowledge related to oyster harvest and ecology^10,48^.

In the San Juan Archipelago on North America’s Northwest Coast, ethnographic accounts provide details on the location of oyster beds, including 11 locations that correspond with 18 archaeological sites that contain oysters, demonstrating continuity between pre- and post-European contact oyster harvest^49^. In southeastern North America, Legus Perryman, Principal Chief of the Muscogee Nation (1875–1895), recounted Muscogee oral history of creation and migration, noting that: “Then they traveled on again eastward until they came to the ocean (‘big water’). They found that the water of the ocean would come up and go out, enabling them to collect oysters and other things good to eat, and they stopped there and lived on those products, being unable to pass beyond”^50^. A few accounts from the Chesapeake in the 1600s note that Kecoughtan tribal members offered John Smith oysters and other foodstuffs in exchange for hatchets and copper, with accounts of feasts during this visit also mentioning oysters^48^. Other accounts from the 17th–18th centuries demonstrate that oysters were roasted by Maryland and North Carolina Algonquians and the Powhatan in Virginia, with these same groups also smoking and drying oysters and some indications of trade of oysters inland^20^. European colonists appropriated land soon after their arrival in Chesapeake Bay, so that Indigenous people had to request permits for limited use of Chesapeake resources that had been freely accessible only one or two generations earlier. According to a 1622 law governing Virginia oyster licenses, “…for the better relief of the poor Indians whom the seating of the English had forced from their wonted convenience of oystering, fishing…, the said justices, shall grant a license to the said Indians to oyster… provided the said justices limit the time the Indians are to stay…”^20,51,52^.

In the Canadian Maritimes, an account from the 1670s suggests Mi’kmaq peoples collected oysters from ice-covered waters in coves near the shoreline in winter^53^. Nicolar, a Penobscot community member in Maine, referenced shell mounds and noted that oysters were mixed with crushed acorns in stews^54^. Additional accounts highlight the importance of oysters to tribes in coastal Maine and New Brunswick, including evidence that oysters were smoked and dried for storage in birch bark containers^55^. These connections continue to more recent times. In the 1970s, Passamaquoddy tribal members set up an oyster farm that enjoyed early success^56^, but was abandoned after a dam that was expected to create warmer waters was never constructed (Donald Soctomah, personal communication January 21, 2021). These accounts exemplify historical and contemporary Indigenous connections to oysters, demonstrate that oysters were parts of local feasts and trade networks, and show that colonists sought to control Indigenous peoples’ access to the oysters they had harvested for thousands of years through the passage of new licensing laws.

### Capitalist commercial fisheries and ecological collapse

Records of oyster harvest after European settlement demonstrate that environmental impacts devastated oyster fisheries in ways that sustained, intensive Indigenous harvests did not. For instance, in Chesapeake Bay, some 17,618,000 bushels of oysters, which is about 1.7 billion individual oysters or 1.1 billion pounds, were harvested in 1891/1892 alone. However, after 1930, the Chesapeake oyster harvest declined to less than 40 million pounds, less than 20 million pounds in the 1980s, and only a few million pounds in the 1990s^57^. Similarly in the Gulf of Mexico, Charlevoix described the coast of Florida in the 1720s as “the dominion of oisters, as the great bank of Newfoundland and the gulf and river of St. Lawrence are that of the cod-fishes.”^58^ In the nineteenth century, Ingersoll noted that beds of oysters were so common on this “low and indented coast” that “each settlement and nearly every farm, as a rule, has its particular locality or bed.”^59^ In 1880, the railway at Cedar Key reportedly hauled away “2710 barrels, equal 6,800 bushels” for sale at cities elsewhere on the Florida Peninsula^59^. But already by this time, many of the beds along the Gulf Coast were said to have been “used up”^59^ or “showing signs of depletion”.^60^

Oyster fisheries on the Northwest Coast of North America suffered similar impacts, but also had to contend with the introduction of non-native species. In Willapa Bay, Washington, the oyster fishery has focused almost entirely on non-native *Crassostrea gigas* (Thunberg 1973) since the early 20th century. Harvest of the native *O. lurida* peaked at around 150,000 bushels of “market-sized” oysters shortly after 1900 with an additional 350,000 bushels of small seed oysters, largely for export to San Francisco Bay where the fishery had already collapsed^15,61^. Today, *O. lurida* has a small and patchy distribution and is considered a species of concern by the state of Washington^61^.

In southern Queensland, Australia, annual harvest of the native *S. glomerata* peaked around 1891 with about 43.8 million harvested oysters, followed by rapid decline and functional disappearance of the fishery. Today, the only notable fishery of *S. glomerata* comes from cultivated oysters in Moreton Bay, where the annual harvest is well under 2 million oysters^62^. Although First Nations Australians were not entirely pushed out of the oyster harvest, they became laborers within the larger capitalist commercial economy. Today, several First Nations groups are trying to reassert their right to harvest oysters, and some families still hold oyster leases established during the late 19^th^ or early 20^th^ century^62,63^.

To the extent that they are discussed at all in ecological studies of oysters, some have characterized Indigenous fisheries as having limited effects on past ecosystems or being “lightly fished,” a description that fails to account for the complexities of a fragmented archaeological record and fundamentally different scales of analysis^16,57,64^. Archaeological and ethnographic data illustrate a range of Indigenous harvest practices from small, seasonally focused camps to large villages with diverse resources, to monuments built for social, political, and religious purposes. At some of these sites, oysters were harvested in the tens of millions or even billions (Table 1). In some cases, archaeological evidence points to use of oyster shells in the construction of artificial reefs for oyster farms^65^. In other cases, such as Chesapeake Bay and southern New England, a large number of smaller sites combine to account for a massive harvest. In the Rhode River, Maryland, for example, four Late Woodland (1100 – 400 BP) sites contained approximately 58 million oysters, but at least 16 other Late Woodland sites with large concentrations of oysters are known to exist in the same small river mouth^66^. It is important to note, in fact, that sites discussed here represent a small fraction of the oysters that people harvested over the past several thousand years. Very few of the known archaeological sites have been fully investigated, and even more sites have been destroyed by sea level rise, coastal development, and mining for road fill or other construction, agricultural uses (e.g., chicken feed supplement), and lime production. Despite the scale of harvest, Indigenous impacts on oyster populations, such as depletion or size declines, were rare and localized^67,68^. Indigenous fisheries were largely sustainable and interwoven with Indigenous people’s worldviews and traditional practices. These fisheries reflected traditional knowledge accumulated by Indigenous peoples over thousands of years that structured technology, harvest practices, and consumption patterns that enabled the incredible yields of the capitalist commercial eras^10,11,48,69,70^.

## Discussion

The millennial-scale archaeological data and sea level rise histories presented here demonstrate that Indigenous oyster fisheries were often massive, providing a crucial food source for people spanning millennia. Pre-colonial nearshore ecosystems were not pristine or “wild”^64^, but hosted fisheries that were woven into the broader traditional marine resource management and cultural systems of local Indigenous peoples, a pattern that is part of the longevity of the fisheries and stands in direct contrast to most capitalist commercial harvest. In fact, Indigenous management often allowed or produced the abundance that was later exploited by settler colonial enterprises. Shell middens and mounds left an indelible legacy on terrestrial landscapes, marking persistent places that are sometimes only clear to Indigenous people and affecting the soil conditions and plant communities found on them today^71,72^.

Oysters are among the most heavily studied marine invertebrates globally, but until recently archaeological data have not been widely incorporated into these research efforts. Capitalist commercial fisheries and environmental change have decimated oyster populations, exacerbating and causing additional ecological collapse^13–18,57,62^. A variety of studies have chronicled the implications of the historical ecology of oyster fisheries for understanding and improving contemporary management and restoration efforts^13,15–17,73^. While a crucial source of information, capitalist commercial harvest records generally only extend back 100–200 years. Recent conservation paleobiology studies of fossil reefs improve the time depth of these studies^8,9^. With few exceptions, however, Indigenous fisheries are a forgotten information source for past fisheries that span thousands of years across a range of environmental and anthropogenic changes. The archaeological record of oyster fisheries contains archives that can help us understand past sea level, salinity, nutrient composition, and climatic changes, and a range of other information. Oysters were an important coastal food source for people in North America and Australia that was managed and maintained for thousands of years, part of a complex web linking local ecologies and people’s identities and worldviews^10,11,48,49^. Large mounds of curated shell stand today as monuments to long-term sustainability.

The current condition of oyster fisheries is not just a question of a baseline shift or an ecological regime shift. In many contexts, oysters were once part of complex socio-ecological systems that no longer exist on the scale they once did, and the ongoing absence of those systems should be considered one of the obstacles to creating healthier, sustainable fisheries. Although there are instances of predation pressure at some localities^67^, our data suggest long-term sustainability of Indigenous oyster fisheries. These Indigenous fisheries were complex, with some focused on local consumption of shellfish combined with hunting, farming, fishing, and exchange, while in others (e.g., the Pacific Northwest) people were producing food for surplus, exchange, and the accumulation of wealth^74^. They were sustained by social relationships, cultural expectations, foodways, and conservation ethics, within societies that treated entire watersheds as interconnected systems rather than separate economic niches (i.e. logging, fishing, farming)^34,36,47,48,65^.

Fisheries collapses are complex phenomena, the result of myriad causes at local to global scales. Often overlooked, though, is the displacement or forcible removal of societies that, based on the data presented here, were part of much healthier ecosystems and supported more sustainable fisheries^75^. Although it often occurred decades or centuries before the fisheries collapses of the 18^th^ to 20^th^ centuries, this displacement was a major factor in unraveling the rest of the system. Conservation biology increasingly recognizes that ecological disasters need to be considered in the context of the colonial atrocities that enabled them. Displacement, moreover, was not a strictly historical event—the ongoing exclusion of peoples with close cultural and ancestral ties to each of the coastal regions discussed here continues to inflict damage on both people and environments and create missed opportunities for more holistic environmental stewardship. Co-production of knowledge with Indigenous groups is not new to fisheries management^2,76,77^, but the records presented here of thousands of years of oyster harvest in the North America and Australia provide an opening and deep time support for Indigenous peoples to restore and broaden their relationships with oysters, and a wide variety of organisms and ecosystems around the world^1,3–5,75,78,79^. Restoration of these relationships could occur in many ways—consultation or co-management between Indigenous and non-Indigenous managers, co-production of knowledge in research, or setting aside separate management regions are just a few examples. While this could have positive benefits for ecosystem and economic health, it would more importantly be a small step towards renewing Indigenous stewardship of their traditional homelands.

## Methods

### Study region and sample selection

Our study draws on datasets from archaeologists and Indigenous scholars from Australia and the Atlantic and Pacific coasts of North America. In Australia, we focus on a region of southeastern Queensland from Bribie Island to Gold Coast City (Fig. 6). We draw on data from 16 shell middens and mounds with robust chronologies and quantified data about oyster abundance. Although early European explorers described extensive oyster reefs of the native Sydney rock oyster (*S. glomerata*) along the coasts of New South Wales and southern Queensland, only a remnant remains and all of the formerly extensive subtidal reefs are gone. Kirby^15^ attributed this largely to overharvest after European colonization. However, Ogburn and colleagues^80^ argue that the European fishery was not significantly larger than the pre-existing Aboriginal fishery, and instead posit that the introduction of a new species, or perhaps multiple species, of mudworm decimated an oyster population weakened by sedimentation and other habitat changes. Although they were once able to live as far as 3 m below the medium low-water mark, increased sedimentation, disease, pests, and pollution currently prevent restoration and aquaculture outside of the narrow intertidal zone^81^.

Along the Pacific coast of North America, we focus on two sub-regions, including 31 archaeological sites in San Francisco Bay in California and 36 archaeological sites in the Salish Sea region of the Pacific Northwest Coast. In both of these regions, the Olympia oyster (*O. lurida*) is the native species harvested by Indigenous peoples before the introduction of the Pacific oyster (*Crassostrea gigas* Thunberg) and other species to increase local fisheries in the mid-19^th^ and early 20^th^ centuries. The Olympia oyster is relatively small, has narrow temperature and salinity tolerances, and is typically found in intertidal areas^82–84^. The combination of limited suitable habitat and competition with the faster growing, introduced Pacific oyster impedes Olympia oyster restoration. The Olympia oyster fishery began to collapse in San Francisco Bay soon after the California gold rush in 1849, followed by successive collapses to the north and south and the extinction of the capitalist commercial fishery by the 1920s^15^. Introduced competitors, predators, pests, and diseases combined with pollution, sedimentation, and freshwater runoff challenge restoration efforts^80,83^.

In eastern North America, the eastern oyster (*C. virginica*) has been widely studied in terms of archaeology^10,11,33^, historical ecology^73,85,86^, and biology^87–89^. We draw on data from 30 sites in New England from the Gulf of Maine to Long Island, 30 sites from Chesapeake Bay, 49 sites from the southern Atlantic coast from South Carolina to Florida, and 15 sites from the Florida Gulf Coast. The eastern oyster can tolerate a wide range of salinity and temperature conditions, is found in waters as deep as 11 m, and is naturally distributed from Canada to Brazil^90,91^. North American fisheries collapsed beginning in and around New York City in the early 19^th^ century, moving southward through the Atlantic and Gulf of Mexico coasts of the United States during the rest of the 19^th^ and early 20^th^ centuries^15^. Although the eastern oyster fisheries produce only a fraction of their potential, they are still among the most robust in the world, and the Gulf of Mexico, in particular, hosts the most extensive wild oyster reefs still in existence^14^.

### Secondary data collection

In each region, we collected data on oyster-bearing sites from primary literature and the authors’ research (Table S1). We included data about (1) site location, (2) site chronology, (3) site type, use, and size, (4) original excavation methods, and (5) oyster abundance within the site. Because methods of analysis differ among different regions and even among sites within the same region, we focused on two measurements: the frequency of oyster bearing sites (Fig. 1) and the relative abundance of oysters within sites (Fig. 3). Researchers collected abundance measurements as the percent of oyster relative to other shellfish measured as either minimum number of individuals (MNI) or weight of shell. Because the two valves of an oyster shell are distinctive (Fig. 1), the MNI can be determined by counting the total number of right and left valves within an analytical unit and using the highest number. Alternatively, some researchers measure shell by weight, and many use both measurements. Although not directly comparable, either is considered an acceptable measurement of relative abundance in zooarchaeological analysis with various strengths and limitations^92–94^.

We also estimated the total number of oysters present for sites where researchers had reported all relevant data—the total site volume, the volume of excavated samples, and the total MNI within those excavated samples^53^. Volume was generally estimated by approximating the site volume based on excavation data and the documented area of sites. In a few cases in the southern Atlantic, LIDAR was used to produce more precise estimates. Finally, for comparative purposes we targeted sites with the same quantitative data across all of the regions. These estimates allow for quantification of the scale and intensity of harvest that can be extrapolated for entire regions. Our data draw on published analyses and reports, gray literature from regional repositories, and personal files, with data sources noted in Table S1. We note, however, that more sites have been excavated and reported on with oysters in each of the regions than we were able to include based on the required data described above.

We surveyed published and unpublished literature for ethnohistoric accounts of oysters among Indigenous communities. To explore relationships between archaeological site function and human activities, we also gathered as much information as possible about archaeological sites. These findings form the basis for interpretations of the cultural, social, and ritual significance of oysters.

### Primary data collection

In some cases, we draw on data provided by coauthors from more recently excavated sites that have not been independently published. These include data from site CA-ALA-307 in the San Francisco Bay region; sites 18AN1640, 18AN1641, 18AN1642, 18AN1644, 44LA149, and several unnamed sites in the Chesapeake Bay region; and sites from Edisto Island, Daws Island, Ossabaw Island, Sapelo Island, and the Indian River in the Southeastern US Atlantic Coast region; and sites from Tampa Bay, Pine Island, Calusa Island, Estero Bay, and Ten Thousand Islands in the Southeastern US Gulf of Mexico region (see Supplementary Data 1). In most of these cases, researchers followed standard techniques for excavation and analysis. Material was excavated from shell mounds or middens in standardized units, ranging in size from 0.25 × 0.25 m column samples to 1 × 2 m units, depending on the size of the site. Material was screened over 3 mm or smaller mesh, with all material retained. Shell and bone were then sorted to the least inclusive taxonomic unit possible. All shellfish were weighed and non-repetitive elements, usually sided hinges, were used to calculate MNI.

The exception is materials from CA-ALA-307, which were analyzed from a museum collection excavated in the 1950’s and housed at the Phoebe Hearst Museum at University of California, Berkeley. Bulk sediments samples of 0.35–1.05 liters from each arbitrary 1-foot level were floated to separate heavy and light fraction materials. Heavy fraction materials were analyzed down to a > 2 mm size fraction. Shell and bone were sorted to the least inclusive taxonomic unit possible using local comparative collections. All shellfish were weighed and non-repetitive elements were used to calculate MNI.

### Indigenous collaboration

The data collected for this paper come from the colonized lands of dozens of Indigenous nations. Modern archaeological research and heritage management in the USA, Canada, and Australia should be performed in consultation and/or collaboration with Indigenous communities. Many of the more recent studies used to form the core of this paper follow this practice, although we acknowledge that many older studies were performed without any consultation. In addition, several of the authors of this paper are members of Indigenous communities from the territories within our study.

### Reporting summary

Further information on research design is available in the Nature Research Reporting Summary linked to this article.

## Supplementary information

Description of Additional Supplementary Files

Supplementary Data 1

Reporting Summary

## Data Availability

All data necessary to perform the analyses described in this paper are available in the Supplementary Data 1. Most of these data are derived from published sources or the authors’ research, as described in the methods section. Data that are not otherwise available come from the US states of Florida, Maine, South Carolina, and Washington. These can be accessed, with permission, from the State Historic Preservation Offices for Florida (https://dos.myflorida.com/historical/), Maine (http://www.state.me.us/mhpc/), South Carolina (https://shpo.sc.gov/), and Washington (https://dahp.wa.gov/). Some data are published with documentation for the National Register of Historic Places (https://www.nps.gov/subjects/nationalregister/index.htm). Source data for Figs. 1 and 3 are provided with this paper. Source data are provided with this paper.

## Source data

Source Data
