# Supplementary material for: Indigenous oyster fisheries persisted for millennia and should inform future management
Source: Nat Commun. 2022 May 3;13:2383. doi: 10.1038/s41467-022-29818-z (PMC9065011; doi:10.1038/s41467-022-29818-z)
Supplement: Supplementary file 1 — Description of Additional Supplementary Files [file 41467_2022_29818_MOESM1_ESM.pdf]

Description of Additional Supplementary Files

Supplementary Data 1: Primary dataset containing information about site age, description, and oyster content.
