# Supplementary material for: Indigenous oyster fisheries persisted for millennia and should inform future management
Source: Nat Commun. 2022 May 3;13:2383. doi: 10.1038/s41467-022-29818-z (PMC9065011; doi:10.1038/s41467-022-29818-z)
Supplement: Supplementary file 3 — Reporting Summary [file 41467_2022_29818_MOESM3_ESM.pdf]

nature portfolio

Corresponding author(s): Leslie Reeder-Myers
Torben Rick

Last updated by author(s): Feb 24, 2022

Reporting Summary

Nature Portfolio wishes to improve the reproducibility of the work that we publish. This form provides structure for consistency and transparency in reporting. For further information on Nature Portfolio policies, see our Editorial Policies and the Editorial Policy Checklist.

Statistics

For all statistical analyses, confirm that the following items are present in the figure legend, table legend, main text, or Methods section.

n/a	Confirmed	
☒	☐	The exact sample size (*n*) for each experimental group/condition, given as a discrete number and unit of measurement
☒	☐	A statement on whether measurements were taken from distinct samples or whether the same sample was measured repeatedly
☒	☐	The statistical test(s) used AND whether they are one- or two-sided<br>*Only common tests should be described solely by name; describe more complex techniques in the Methods section.*
☒	☐	A description of all covariates tested
☒	☐	A description of any assumptions or corrections, such as tests of normality and adjustment for multiple comparisons
☒	☐	A full description of the statistical parameters including central tendency (e.g. means) or other basic estimates (e.g. regression coefficient) AND variation (e.g. standard deviation) or associated estimates of uncertainty (e.g. confidence intervals)
☒	☐	For null hypothesis testing, the test statistic (e.g. *F*, *t*, *r*) with confidence intervals, effect sizes, degrees of freedom and *P* value noted<br>*Give P values as exact values whenever suitable.*
☒	☐	For Bayesian analysis, information on the choice of priors and Markov chain Monte Carlo settings
☒	☐	For hierarchical and complex designs, identification of the appropriate level for tests and full reporting of outcomes
☒	☐	Estimates of effect sizes (e.g. Cohen's *d*, Pearson's *r*), indicating how they were calculated

Our web collection on statistics for biologists contains articles on many of the points above.

Software and code

Policy information about availability of computer code

Data collection	No software was used in data collection.
Data analysis	We used Microsoft Excel 2016 for data analysis.

For manuscripts utilizing custom algorithms or software that are central to the research but not yet described in published literature, software must be made available to editors and reviewers. We strongly encourage code deposition in a community repository (e.g. GitHub). See the Nature Portfolio guidelines for submitting code & software for further information.

Data

Policy information about availability of data

All manuscripts must include a data availability statement. This statement should provide the following information, where applicable:

- Accession codes, unique identifiers, or web links for publicly available datasets
- A description of any restrictions on data availability
- For clinical datasets or third party data, please ensure that the statement adheres to our policy

All data necessary to perform the analyses described in this paper is available in the Supplementary Information. Most of these data are derived from published sources, as cited, or the authors' research, as described in the methods section. We use some small datasets that are neither available in the published literature nor described in this paper. These can be accessed, with permission, from the State Historic Preservation Offices for Florida (https://dos.myflorida.com/historical/), Maine (http://www.state.me.us/mhpc/), South Carolina (https://shpo.sc.gov/), and Washington (https://dahp.wa.gov/). Some data are published with documentation for the National Register of Historic Places (https://www.nps.gov/subjects/nationalregister/index.htm). We have provided source data for figures 1 and 3.

Field-specific reporting

Please select the one below that is the best fit for your research. If you are not sure, read the appropriate sections before making your selection.

☐ Life sciences ☐ Behavioural & social sciences ☒ Ecological, evolutionary & environmental sciences

For a reference copy of the document with all sections, see nature.com/documents/nr-reporting-summary-flat.pdf

Ecological, evolutionary & environmental sciences study design

All studies must disclose on these points even when the disclosure is negative.

Study description	This study examines oyster harvest by Indigenous people in North America and Australia, using measurements of oyster abundance from archaeological sites, ethnohistory, ethnography, and Indigenous knowledge.
Research sample	The research sample includes all archaeological sites known to contain oyster shell within seven regions--southeast Queensland, Australia, the Pacific Northwest of North America, San Francisco Bay, New England, Chesapeake Bay, the southeastern Atlantic Coast of North America, and the Peninsular Gulf Coast of Florida. Data were derived from the published literature, government data repositories, and the authors' own research. The sample was chosen to correspond to areas considered by a previous paper (Kirby, M. X. Fishing down the coast: Historical expansion and collapse of oyster fisheries along continental margins. Proceedings of the National Academy of Sciences 101, 13096 (2004)), in order to expand on that study's goals of examining oyster historical ecology.
Sampling strategy	Each authors created exhaustive lists of archaeological sites that contained oyster within their region of expertise, using published papers, dissertations or other academic productions, and government data repositories. Coauthors identified all sites that contained any amount of oyster and that had reliable chronological information, which formed the dataset for Figure 1. We then narrowed the sample down to sites that had quantified faunal data such that we could determine how much oyster was present relative to other shellfish (either by weight or by minimum number of individuals) . These formed the dataset for the analysis in Figure 2. Finally, we narrowed to a still smaller group of sites where we could estimate the total amount of oyster shell harvested by Indigenous people, which required an estimate of the volume of the entire site, the amount of oyster per excavation unit, and the volume of the excavation unit. The results of this analysis are presented in Table 1. We note, however, that archaeological samples are biased by a number of factors, including site preservation, research priorities, and land ownership.
Data collection	Authors entered data for their region into a standardized spreadsheet, a modified version of which is now represented by the supplemental data table.
Timing and spatial scale	The spatial scale matches that of irby, M. X. Fishing down the coast: Historical expansion and collapse of oyster fisheries along continental margins. Proceedings of the National Academy of Sciences 101, 13096 (2004).
Data exclusions	Sites were excluded if they had confirmed oyster but unreliable or uncertain chronological information.
Reproducibility	There are no experimental findings.
Randomization	There are no experimental findings, and randomization is generally not feasible with archaeological research because samples are determined by the history of site preservation and research.
Blinding	There are no experimental findings, and blinding is generally not feasible with archaeological research.

Did the study involve field work? ☐ Yes ☒ No

Reporting for specific materials, systems and methods

We require information from authors about some types of materials, experimental systems and methods used in many studies. Here, indicate whether each material, system or method listed is relevant to your study. If you are not sure if a list item applies to your research, read the appropriate section before selecting a response.

Materials & experimental systems

n/a	Involved in the study	
☒	☐	Antibodies
☒	☐	Eukaryotic cell lines
☒	☐	Palaeontology and archaeology
☒	☐	Animals and other organisms
☒	☐	Human research participants
☒	☐	Clinical data
☒	☐	Dual use research of concern

Methods

n/a	Involved in the study	
☒	☐	ChIP-seq
☒	☐	Flow cytometry
☒	☐	MRI-based neuroimaging
